# TGFβ, smooth muscle cells and coronary artery disease: a review

**DOI:** 10.1016/j.cellsig.2018.09.004

**Published:** 2019-01

**Authors:** Emma L. Low, Andrew H. Baker, Angela C. Bradshaw

**Affiliations:** a Institute for Cardiovascular and Medical Sciences, University of Glasgow, 126 University Place, Glasgow G12 8TA, UK; b Queen’s Medical Research Institute, University of Edinburgh, 47 Little Crescent, Edinburgh EH16 4TJ, UK

**Keywords:** Transforming growth factor-beta, Smads, Cardiovascular disease, Smooth muscle cells, Vascular cells, Revascularisation surgery

## Abstract

Excessive vascular smooth muscle cell (SMC) proliferation, migration and extracellular matrix (ECM) synthesis are key events in the development of intimal hyperplasia, a pathophysiological response to acute or chronic sources of vascular damage that can lead to occlusive narrowing of the vessel lumen. Atherosclerosis, the primary cause of coronary artery disease, is characterised by chronic vascular inflammation and dyslipidemia, while revascularisation surgeries such as coronary stenting and bypass grafting represent acute forms of vascular injury. Gene knockouts of transforming growth factor-beta (TGFβ), its receptors and downstream signalling proteins have demonstrated the importance of this pleiotropic cytokine during vasculogenesis and in the maintenance of vascular homeostasis. Dysregulated TGFβ signalling is a hallmark of many vascular diseases, and has been associated with the induction of pathological vascular cell phenotypes, fibrosis and ECM remodelling. Here we present an overview of TGFβ signalling in SMCs, highlighting the ways in which this multifaceted cytokine regulates SMC behaviour and phenotype in cardiovascular diseases driven by intimal hyperplasia.

## Highlights

• Dysfunctional smooth muscle cells (SMCs) contribute to the development of intimal hyperplasia and coronary artery disease (CAD)
• Transforming growth factor-beta (TGFβ) is a powerful regulator of SMC phenotype and function
• The activities of TGFβ in CAD are context-dependent, with both atheroprotective and atherogenic roles in SMCs
• TGFβ regulates SMC proliferation and ECM secretion after revascularisation surgery
• The TGFβ signaling axis is an attractive target for therapies aimed at reducing SMC dysfunction and fibrosis in CAD

## 1 Introduction

Classic ultrastructural studies by Schwartz *et al* were the first to show the presence of morphologically identifiable vascular smooth muscle cells (SMCs) migrating though the internal elastic lamina following acute vascular injury in a rat model of balloon angioplasty [1]. Later, seminal work by Clowes *et al* using [^3^H]-thymidine labelling showed that over 40% of medial SMCs were actively proliferating 48 hours post-injury, indicating that a large proportion of SMCs within the vascular wall retain the capacity to re-enter the cell cycle and contribute to vascular remodelling and repair in adult animals [2]. This phenotypic plasticity of SMCs is now understood to play a significant role in the development of intimal hyperplasia, a pathological vascular remodelling process that occurs during the development of coronary artery disease following prolonged exposure to dyslipidaemia, hypertension and inflammation [[3], [4], [5]] or as a consequence of revascularisation surgery, such as coronary artery bypass grafting (CABG) or percutaneous coronary intervention (PCI) [6]. In the latter, a combination of ischemic-reperfusion injury, acute physical damage and increased longitudinal and circumferential shear stress results in endothelial cell (EC) activation, triggering the release of cytokines and growth factors, including transforming growth factor-beta (TGFβ) [7]. In concert, these growth factors and cytokines drive the de-differentiation of quiescent ‘contractile’ SMCs into an active ‘synthetic’ state, in which they display enhanced proliferation, migration and secretory capacity [6].

## 2 The TGFβ signalling pathway

TGFβ is the prototype of the highly-conserved TGFβ superfamily, members of which are potent regulators of SMC phenotype and function in vascular homeostasis and disease [8]. TGFβ superfamily share the same overall structure, consisting of two extended monomers held together by an intermolecular disulphide bond [9]. All TGFβ monomers incorporate a characteristic ‘cysteine knot’ structure, composed of three intramolecular disulphide bonds linking six conserved cysteine residues [10]. Three TGFβ isoforms are expressed in mammals (TGFβ 1-3) and are differentially localised in major blood vessels during development, with TGFβ1 highly localised to the tunica intima, TGFβ2 restricted to the tunica media and TGFβ3 expressed throughout the whole vessel wall [11,12]. In adults, TGFβ1 and TGFβ3 proteins are mainly localised to the arterial intima, with TGFβ1 present in around 50% of the intimal stellate-shaped SMC population [13]. TGFβ is secreted as part of a large latent complex (LLC), consisting of the C-terminal mature TGFβ peptide and N-terminal latency associated peptide (LAP) covalently bound to large latent TGFβ binding proteins (LTBP) [14]. LTBPs stabilise latent TGFβ complexes and facilitate their retention at the cell surface through direct interactions with fibrillin and other ECM proteins [15], while RGD sequences in the LAP target latent TGFβ to integrin receptors [16]. Activation of latent TGFβ at the cell surface is induced primarily by proteases such as furin and plasmin, which cleave the covalently-bound LAP-LTBP pair from the mature TGFβ molecule [17]. Proteolytic cleavage of LAP-LTBP yields short-lived, biologically active TGFβ homodimers which are able to interact with transmembrane TGFβ type III receptors such as betaglycan (also known as TβRIII) and endoglin [18]. Betaglycan is expressed in the majority of cell types, whereas endoglin is most abundantly expressed in vascular ECs, although recent studies have also shown localisation to SMCs in diseased vessels [[19], [20], [21], [22]]. Both betaglycan and endoglin are now thought to have important cellular functions beyond their actions as TGFβ co-receptors, which are reviewed at length elsewhere [23,24].

**Fig. 1:**
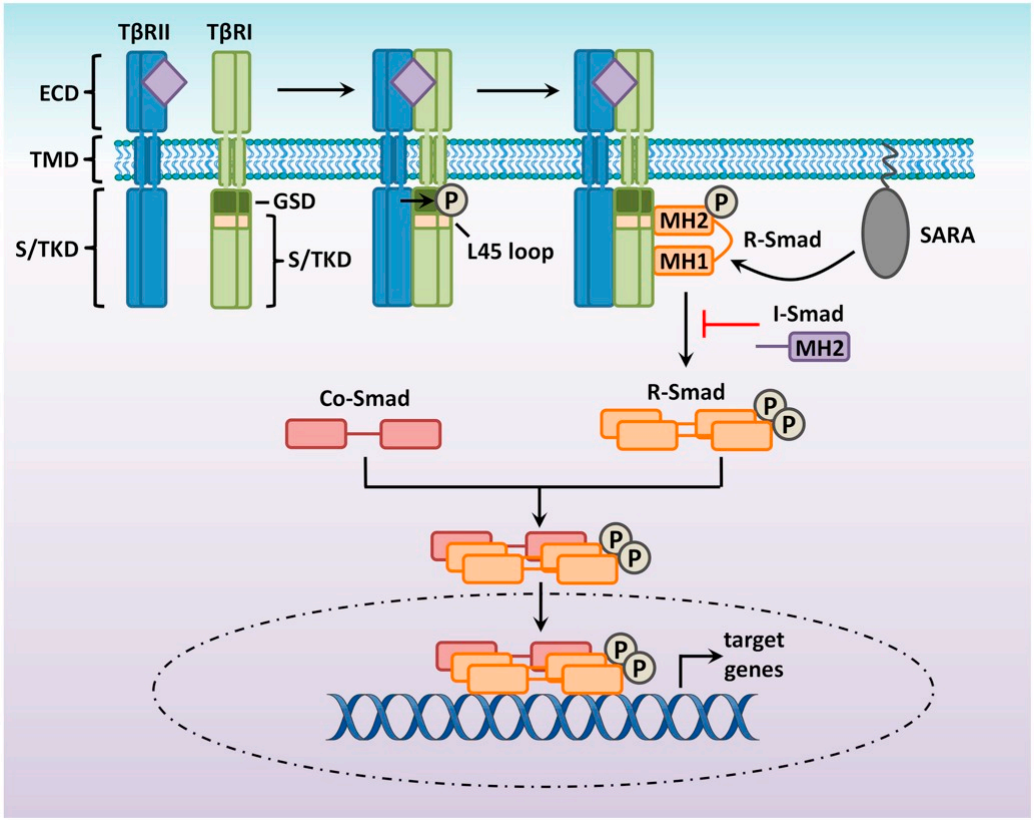
- Canonical TGFβ signalling pathway. Active TGFβ homodimers signal via binding to specific transmembrane receptor complexes comprised of two type I (TβRI) and two type II (TβRII) serine/threonine kinase receptors. TβRI and TβRII are structurally similar with small cysteine-rich extracellular domains (ECD), single transmembrane domains (TMD) and highly conserved intracellular serine/threonine domains (S/TKD). TGFβ binding to TβRII induces the assembly of TβRII and TβRI receptors into a heteromeric complex, within which constitutively active TβRII phosphorylates TβRI at several serine and threonine residues within its conserved glycine-serine domain (GSD). R-Smads become phosphorylated by the activated TβRI at their C-terminal SSXS motif. The L45 loop of TβRI and the L3 loop of the R-Smad MH2 domain determine R-Smad receptor specificity, with ALK5 specifically phosphorylating Smads 2 and 3. The adaptor protein Smad anchor for receptor activation (SARA) can also facilitate recognition of R-Smads by the receptors. I-Smads contain MH2 domains and can act to turn off Smad TGFβ signalling by interfering with Smad-receptor or Smad-Smad interactions. Phosphorylated R-Smads form a heteromeric complex with Co-Smad, accumulate in the nucleus and directly regulate the transcription of specific target genes.

Binding to type III accessory receptors facilitates TGFβ signalling through presentation of ligand to signal transduction receptors at the cell surface. Active TGFβ homodimers signal via specific transmembrane heteromeric complexes comprised of two type I and two type II serine/threonine kinase receptors [25]. Five TGFβ superfamily type II receptors and seven type I receptors exist in mammals [26]. The type I and type II receptors are structurally similar with small cysteine-rich ECDs (100-140 amino acids), single TMDs (30-35 amino acids) and highly conserved intracellular serine/threonine kinase domains (S/TKD; 350-400 amino acids) [9]. Each member of the TGFβ superfamily binds to a characteristic combination of type I and type II receptors (Table 1). Analysis of the crystal structures of TGFβ ligand:receptor ternary complexes has revealed that the length and conformation of the ligand fingertips and receptor ligand binding loops are important determinants of ligand: receptor specificity [27]. These studies have illustrated that TGFβ ligands use their conserved Site IIa in their fingertip region to bind the β1 and β2 strands within the ECD of the TGFβ type II receptor (TβRII) [28]. Importantly, the β4-β5 region within the ECD of the TβRII contains a 5-8 amino acid insertion which ensures type II receptor specificity by blocking binding of TβRII to bone morphogenetic protein (BMP) ligands. Of the five mammalian type II receptors, TGFβ binds specifically to TβRII (also known as TGFBR2), which is highly expressed throughout the intima and media of adult vessels [13]. Early membrane crosslinking studies confirmed the expression of TβRII in SMCs, also showing binding of I^125^TGFβ1 to receptor complexes composed of type I, II and III TGFβ receptors [29]. TβRII ligand binding induces the assembly of type I and type II receptors into a heteromeric complex, within which constitutively active TβRII phosphorylates type I receptors at several serine and threonine residues within their conserved glycine-serine (GS) domains [8,30]. TGFβ ligands principally signal via activin receptor-like kinase 5 (ALK5, a type I receptor also known as TβRI) [14]. In addition to ALK5, TGFβ can also signal via another type I receptor called activin receptor-like kinase 1 (ALK1), via a distinct Smad-mediated signalling pathway to ALK5 [[31], [32], [33], [34], [35]]. While ALK5 is predominantly expressed in medial SMCs in vessels from healthy adult animals, ALK1 is chiefly localised to the endothelium, although it is upregulated in SMCs following acute vascular injury or during atherogenesis [[36], [37], [38]]. Following activation, type I TGFβ receptors propagate the signal inside the cell through activation of the canonical Smad signalling pathway, as well as other Smad-independent kinase pathways (Fig. 1; [25]). Readers are directed to a series of excellent reviews on TGFβ signalling via non-canonical kinase pathways [26,39,40].

**Table 1:** - Ligands, receptors and R-Smads in the TGFβ superfamily

Ligand	Type I receptor	Type II receptor	Type III receptor	R-Smad
TGFβ1 TGFβ2 TGFβ3	ALK1/5	TβRII	Betaglycan Endoglin	Smad1/5/8 Smad2/3
BMP2 BMP4	ALK3/6	BMPRII	RGM Betaglycan/Endoglin	Smad1/5/8
BMP5 BMP6 BMP7	ALK2/3/6	BMPRII ActRIIA ActRIIB	Betaglycan Endoglin	Smad1/5/8
BMP8A BMP8B	ALK3/5	BMPR2/ActRIIA ActRIIB/TβRII	Not known	Smad1/5/8 Smad2/3
BMP9 BMP10	ALK1/3/6	BMPRII/ActRIIA	Endoglin	Smad1/5/8
GDF7 GDF6 GDF5	ALK2/3/6	BMPRII/ActRIIA/ ActRIIB	Not known	Smad1/5/8
AMH	ALK2/3/6	AMHRII	Not known	Smad1/5/8
Activin A/AB/B GDF8 GDF11	ALK4	BMPRII ActRIIA ActRIIB	Betaglycan Endoglin	Smad2/3
BMP16/Nodal	ALK7	BMPRII/ActRIIA ActRIIB	Not known	Smad2/3

TGFβ = transforming growth factor beta, BMP = bone morphogenetic protein, GDF = growth/differentiation factor, AMH = anti-Mϋllerian hormone, RGM = repulsive guidance molecule

## 3 Canonical Smad TGFβ signalling

Smad proteins are the principal intracellular mediators of TGFβ superfamily signalling. Of the eight Smad proteins expressed in mammals (Smads 1-8), Smads 2 and 3 are the primary receptor-regulated Smads (or R-Smads) activated by receptors for the three TGFβ ligands [25,41]. Smad4, also known as Co-Smad, serves as a common partner for all R-Smads. Smad6 and Smad7 are inhibitory Smads (I-Smads) which act to turn off Smad TGFβ signalling by interfering with Smad-receptor or Smad-Smad interactions [25]. In general, all Smads are widely expressed throughout development and in adult animals [42]. The R-Smads and Co-Smad share homologous N- and C-terminal regions, called the Mad-homology 1 (MH1) and MH2 domains respectively, separated by a divergent proline-rich linker region [43]. I-Smads contain conserved MH2 domains but do not possess MH1 domains [8]. With the exception of Smad2, the MH1 domains of Smads exhibit sequence specific DNA binding activity, whereas MH2 domains mediate Smad oligomerisation and Smad-receptor interactions [25,44]. The linker region of R-Smad contains multiple phosphorylation sites which allow specific crosstalk with other signalling pathways including mitogen-activated protein kinases (MAPKs) and cyclin-dependent kinases, and a PY motif which mediates specific interactions with the Smurf ubiquitin ligases [25].

In non-stimulated cells, Smads undergo a constant process of nucleocytoplasmic shuttling, with the rate of nuclear export being higher than the rate of import, such that the R-Smads are predominantly localised to the cytoplasm [45]. In contrast, I-Smads tend to be localised within the nucleus in non-stimulated cells and Smad4 is distributed equally between both compartments [46]. Upon ligand stimulation, R-Smads become phosphorylated by the activated type I receptor at their C-terminal SSXS motif, which increases their affinity for Smad4 [25]. The L45 loop of the type I receptor (located adjacent to its GS region) and the L3 loop of the R-Smad C-terminal domain determine R-Smad receptor specificity. The primary TGFβ type I receptor in SMCs, ALK5, specifically phosphorylates Smads 2 and 3 [47,48]. Receptor recognition of R-Smads can be facilitated by auxiliary proteins, such as the adaptor protein, Smad anchor for receptor activation (SARA). SARA contains a phospholipid binding FYVE domain which targets Smads 2 and 3 to the plasma membrane and early endosomes, where it facilitates their interaction with the activated TβRI [49]. Phosphorylated R-Smads form a heteromeric complex with Smad4 and accumulate in the nucleus following importin-mediated nuclear translocation [25].

Nuclear R-Smad/Smad4 complexes bind directly to Smad-binding elements (SBE) in the promoters of TGFβ target genes via a highly conserved β-hairpin loop within their MH1 domain [50]. Although many Smad-responsive promoter regions contain one or more SBEs [50], oligonucleotide binding assays have shown that Smad complexes can also recognise and bind GC-rich promoter sequences, demonstrating a relaxed DNA-binding specificity of the Smad MH1 domain [41]. As the affinity of Smad binding to a single SBE is insufficient to support sustained binding to DNA in the absence of co-operating transcriptional partners [50,51], they exert the majority of their effects on gene expression in co-operation with DNA binding co-factors, co-activators and co-repressors [41]. For example, the transcription factor δEF1 (also known as ZEB-1) is selectively expressed in SMCs and transactivates the promoters of SMC differentiation markers following TGFβ1 stimulation of SMCs, by directly binding Smad3 and serum response factor (SRF) [52]. Similarly, the transcriptional coactivator myocardin physically associates with Smad3 in SMCs, co-ordinately transactivating the promoters of the *SM22α*, smooth muscle myosin heavy chain (*SMMHC*) and smooth muscle α-actin genes (*ACTA2*; [53]). Thus, while Smad proteins are ubiquitously expressed, the expression of Smad transcriptional partners is generally restricted to certain cell types, thereby providing a mechanism for cell lineage-specific gene responses [41]. Readers are directed to two excellent recent reviews on the contextual control of gene transcription elicited by Smad proteins [54,55].

## 4 TGFβ in coronary artery disease

**Fig. 2:**
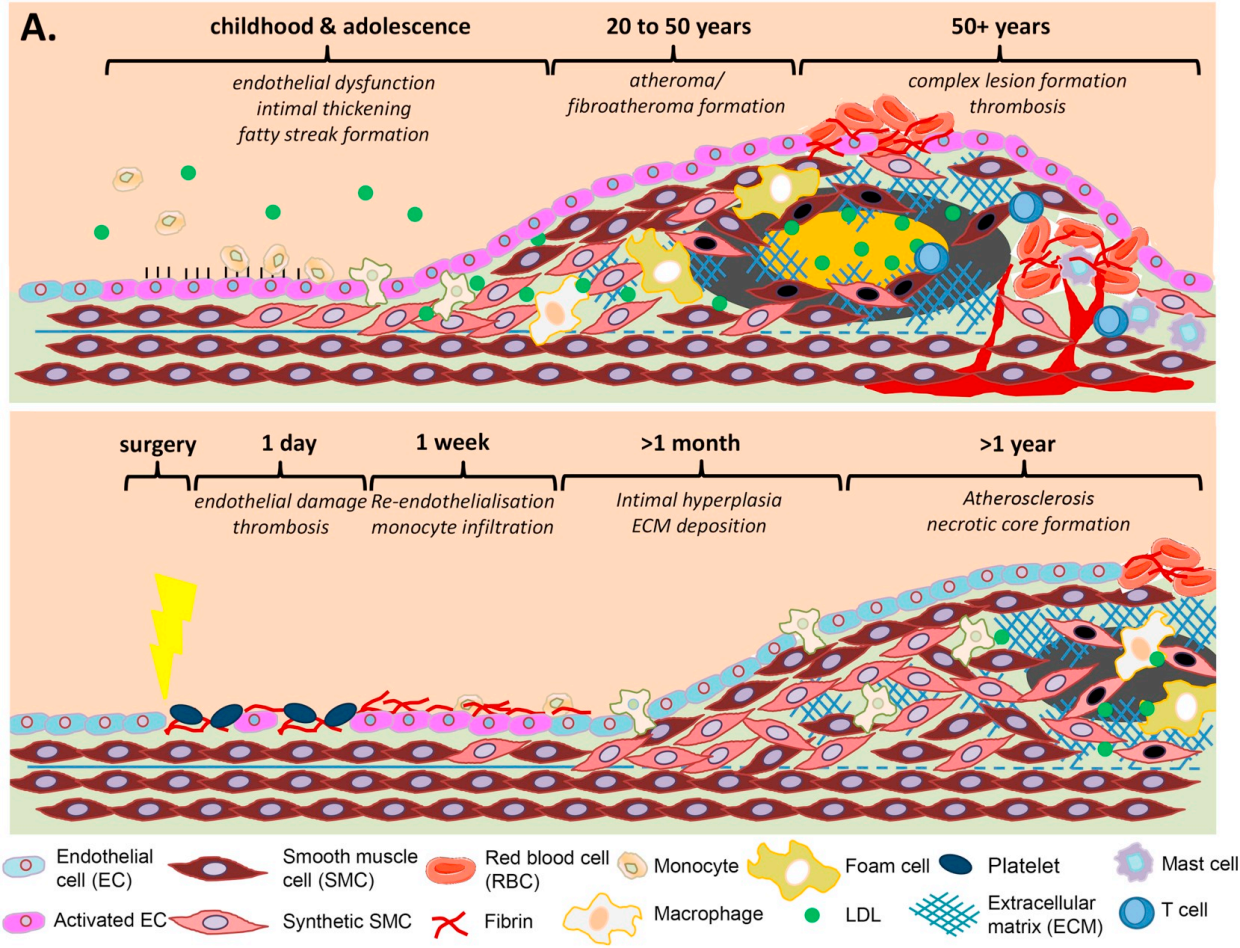
– Vascular remodelling during atherosclerosis (A) and after revascularisation surgery (B) (A) Atherosclerosis is initiated by the activation of the endothelium in response to oxidative, haemodynamic or biochemical stimuli. Activated endothelial cells (ECs) upregulate surface adhesion molecules and secrete growth factors and cytokines, promoting rolling adhesion of circulating leukocytes as well as activation of the underlying smooth muscle cells (SMCs). Activated SMCs dedifferentiate and start proliferating and migrating, contributing to the growing neointima. Leukocytes adhering to the endothelium migrate into the intima through diapedesis, maturing into macrophages and phagocytosing low density lipoproteins to become foam cells, characteristic of the ‘fatty streak’ lesions that can be observed from adolescence onwards. Fibroatheromas form from areas of intimal thickening, which consist of foam cells, remnants of apoptotic SMC and a lipid rich ECM pool. Early fibroatheromas are characterised by an acellular necrotic core and a thick fibrous cap, composed of collagen fibrils interspersed with SMCs. Advancing fibroatheromas contain cholesterol crystals, neovessels and lymphocytes, and have thin fibrous caps due to proteolytic ECM degradation, making these lesions particularly susceptible to rupture and thrombosis. Rupture and thrombosis frequently occurs at the shoulder regions of plaques, where mast cells accumulate and secrete pro-angiogenic factors and enzymes to further promote microvessel formation. (B) Vein graft implantation or coronary stent deployment induces endothelial damage and denudation. Within hours, platelets and red blood cells adhere to the endothelial layer, initiating a coagulation cascade that results in the deposition of fibrin-rich layers. In the weeks following surgery, circulating leukocytes attach and infiltrate the vascular endothelium, while SMCs in the media are activated and start migrating into the growing neointima. Growth factors and cytokines released by cells in the vessel wall induce SMC proliferation and ECM deposition, resulting in further intimal thickening and inward vascular remodelling. Intimal thickening can act as a substrate for superimposed atherosclerosis or neoatherosclerosis, which is frequently observed between 2-5 years following revascularisation surgery. The pathogenesis of superimposed atherosclerosis/neoatherosclerosis bears many similarities with native coronary artery atherosclerosis (A), albeit within a much shorter timeframe.

Coronary artery disease (CAD) is primarily caused by atherosclerosis, which leads to the formation of occlusive, lipid-rich plaques in affected vessels (Fig. 2A) [56]. Prolonged exposure to cardiovascular risk factors such as dyslipidemia, hypertension and inflammation promotes endothelial dysfunction, which precedes atherosclerotic lesion formation [[3], [4], [5]]. The increased vascular permeability of dysfunctional, activated endothelial cells (ECs) promotes the entry of low density lipoproteins (LDLs) from the circulation into the vascular intima. Proteoglycans in the arterial wall (such as versican, biglycan and decorin) bind and retain LDLs, which become oxidised (oxLDL; [[57], [58], [59]]). OxLDL induces the secretion of chemokines and the expression of leukocyte adhesion molecules, which together promote monocyte infiltration into the sub-endothelial space [60]. Within the intima, SMC- and EC-derived cytokines induce monocytes to differentiate into macrophages that engulf oxLDL, forming foam cells. In turn, inflammatory cells within the early lesion secrete cytokines and growth factors which promote the development of intimal hyperplasia. Resident SMCs are key drivers of intimal hyperplasia in the initiation and early progression of atherosclerosis, which is characterised by SMC dedifferentiation, proliferation and migration [61]. Secretory SMCs synthesise an abundant array of ECM components, which form a fibrous cap over the plaque, further encroaching on the vessel lumen [62]. Increased synthesis of proteoglycans by secretory SMCs also promotes lipoprotein retention in the growing lesions, while dedifferentiated SMCs acquire phenotypic characteristics of the osteoblast, adipocyte and macrophage lineages [63]. Advanced, rupture-prone plaques are characterised by lipid-rich necrotic cores (composed of apoptotic foam cells and cellular debris) thin fibrotic caps (a consequence of matrix metalloproteinase secretion), vascular calcification and neoangiogenesis.

Several genome-wide association studies (GWAS) have identified an association between CAD and single nucleotide polymorphisms (SNPs) in genes encoding TGFβ signalling pathway components. For example, functional polymorphisms in the promoter, signal peptide sequence and coding sequence of the *TGFβ1* gene are associated with increased risk of myocardial infarction [64,65]) and stroke [66]; meta-analyses have also shown an association between these polymorphisms and CAD [[67], [68], [69], [70]]. In addition, a joint analysis of two GWAS on CAD patients identified an association with an intronic SNP in the *SMAD3* gene [71] which was later shown to reduce enhancer activity and attenuate Smad3 expression [72,73]. Clinical studies have illustrated that plasma levels of active TGFβ1 are markedly reduced in patients with advanced atherosclerosis compared with healthy controls [74,75]. In contrast, other groups have reported an increase in active TGFβ1 levels in the plasma of CAD patients, where patients with triple vessel disease had twice the level of circulating active TGFβ1 compared with those with no or mild CAD [76]. These differences may be due to discrepancies between sample preparation methods, which can affect the level of TGFβ1 protein detected in plasma [77]. Nevertheless, immunolocalisation studies of human atherosclerotic lesions strongly support a role for TGFβ in the pathogenesis of CAD, showing high levels of TGFβ1 and TGFβ3 in SMCs and macrophage-derived foam cells in early fatty streak lesions, co-localising with TβRII and ALK5 [13]. >50% of SMCs in early lesions also stain positive for phospho-Smad2, indicative of TGFβ signalling pathway activation in these cells [78]. Advanced fibrous lesions also express significant amounts of TGFβ1, whereas TGFβ3, TβRII and ALK5 are expressed at more reduced levels in the fibrous plaque and the associated media [13].

Studies in experimental models of atherosclerosis indicate that TGFβ can be both atheroprotective and atherogenic. Early animal studies using global TGFβ inhibition or genetic knockout approaches demonstrated that reduced availability of TGFβ was pro-atherogenic and associated with the development of macrophage-rich, pro-inflammatory plaques which were prone to rupture [79]. Mice heterozygous for the deletion of *tgfb1* on a cholesterol-enriched diet had reduced SMC differentiation (determined by levels of αSMA and SMMHC, two mature SMC marker proteins), accelerated lipid lesion formation and increased vascular inflammation compared with wild-type littermate controls [80]. Similarly, administration of a neutralising anti-TGFβ1 antibody [81], or systemic infusion of a dominant negative TβRII in apolipoprotein E (ApoE)-deficient mice [82] significantly enhanced lipid infiltration in the vascular wall, decreased collagen type I and III secretion by SMCs and was associated with frequent intraplaque haemorrhages. Corroborating these TGFβ knockdown studies, overexpression of an activated TGFβ1 expression construct via viral gene transfer markedly reduced atherosclerotic lesion formation in fat-fed LDL receptor knockout mice [83]. In these animals, medial and intimal SMCs showed reduced expression of the oxidative stress marker nitrotyrosine, with CD68+ macrophage infiltration also substantially attenuated as a result of diminished SMC-derived M-CSF secretion [83]. Similarly, overexpression of active TGFβ1 in the hearts of ApoE^-/-^ mice reduced aortic root plaque formation by decreasing inflammatory cell infiltration and increasing SMC collagen secretion to form more stable atherosclerotic lesions [84]. Interestingly, pre-incubation of rat SMCs with atorvastatin enhanced the TGFβ1-mediated activation of Smad2/3; similar results were observed in ApoE-/- mice treated with a moderate dose of statin, accompanied by increased collagen and αSMA staining in plaques [85]. Together, these studies support the ‘protective cytokine’ theory of atherosclerosis [86], indicating that TGFβ can protect against the development of unstable plaque lesions by promoting the expression of contractile SMC proteins, supressing leukocyte recruitment, and reinforcing the fibrous cap by enhancing ECM production by resident SMCs.

There is, however, an important caveat to these observations; while the induction of contractile marker proteins (such as αSMA and SM22α) by TGFβ can be viewed as atheroprotective in SMCs, very recent studies investigating the origin of αSMA+ cells within atherosclerotic lesions have demonstrated that activation of this transcriptional programme by TGFβ in endothelial cells (ECs) can instead promote the induction of atherosclerosis. Using endothelial lineage tracing mice on an ApoE^-/-^ background (*SclCreER*^T2^; *R26RstopYFP*;*ApoE*^*−/−*^), Evrard *et al* found that TGFβ could induce endothelial-to-mesenchymal transition (EndMT) during atherogenesis, enhancing expression of αSMA and fibrotic markers in ECs without affecting collagen expression (87). Of note, immunohistological evaluation of human atherosclerotic lesions revealed a higher proportion of cells co-expressing endothelial and fibroblast markers in type VI plaques (complicated lesions with unstable features) compared to type V plaques (stable fibrocalcific lesions/fibroatheromas) supporting a role for TGFβ-induced EndMT in the clinical context [87]. There is also accumulating evidence that TGFβ can elicit atherogenic effects through its actions on SMCs in early plaque lesions. For example, while the promotion of contractile protein expression in SMCs is an important part of TGFβ’s anti-atherogenic repertoire during the later stages of plaque development, increased vascular resistance and SMC hypercontractility is also associated with the induction of atherosclerosis [88]. Additionally, TGFβ is now known to be a potent inducer of proteoglycan (PG) synthesis by SMCs, enhancing the gene expression and glycosaminoglycan (GAG) sidechain elongation of PGs such as biglycan [89,90] and versican [91]. PGs directly contribute to the initiation of atherosclerosis through their electrostatic interactions with lipoproteins, promoting the retention of lipoproteins in the sub-endothelial space (reviewed in [92]). Accordingly, treatment of atheroprone LDLr^-/-^ mice with the TGFβ neutralising antibody 1D11 substantially repressed biglycan expression, reducing biglycan colocalisation with apoB lipoproteins and attenuating atherosclerotic lesion formation [93]. Finally, it has recently been recognised that TGFβ can drive the transdifferentiation of SMCs into proliferative, αSMA-positive migratory myofibroblasts, thereby contributing to the early development of atherosclerotic plaques, whilst on the other hand promoting stability of more advanced lesions through fibrotic cap formation [94,95]. Thus, while TGFβ generally acts as a potent pro-fibrotic and anti-inflammatory mediator in CAD, the pathophysiological outcome of these actions is highly context-dependent, varying according to the specific cell type, stage of atherosclerosis (early/advanced) and type of lesion (stable/unstable).

## 5 TGFβ in acute vascular injury: vein graft failure and restenosis

Revascularisation surgeries such as percutaneous coronary intervention (PCI) or coronary artery bypass grafting (CABG) are frequently prescribed for advanced or acute presentations of CAD, aiming to widen occluded coronary arteries. However, the long-term patency of such procedures is hampered by the development of intimal hyperplasia within the vessel, resulting in re-occlusion and the need for repeat intervention. Similar to the intimal hyperplasia (IH) that forms a fertile ‘soil’ for atherosclerosis in CAD, IH following revascularisation surgery is initiated by activation of the endothelium. Stent deployment (PCI) or exposure of venous bypass conduits to increased arterial shear stress (CABG) induces acute endothelial injury, leading to adhesion of circulating platelets and monocytes to the endothelium. Pro-inflammatory growth factors and cytokines released by activated endothelial cells, platelets and leukocytes drive SMC dedifferentiation, proliferation and migration, cellular processes that are critical to the development of IH. Medial SMC proliferation is rapidly induced following vascular injury and peaks around 7 days post-injury (10 - 20 % medial SMC proliferation; [96] [97]). Proliferating medial SMCs also migrate and accumulate in the intima, contributing to the overall lesion cell mass [98,99]. The resulting hyperplastic neointima can act as a substrate for accelerated atherosclerotic plaque formation, which further contributes to the occlusion of the vessel. Compared to native CAD, which takes decades to develop, vein graft atherosclerosis develops over a period of months to a few years. Lesions are also more concentric and diffuse than native atherosclerotic lesions and are more susceptible to thrombosis and rupture ([100,101]; reviewed in [102]). There is growing awareness that in-stent atherosclerosis (most frequently termed ‘neoatherosclerosis’) is one of the primary causes of late stent failure, with several studies showing that the development of neoatherosclerosis is accelerated in drug-eluting stents (DES) compared to first-generation bare-metal stents (BMS) [[103], [104], [105], [106]]. Although the mechanisms causing neoatherosclerosis have yet to be fully characterised, histopathological and intravascular imaging studies indicate that stent-induced shear stress, chronic inflammation and endothelial dysfunction may be key contributing factors (Fig. 2B) [102].

Clinical studies of restenotic arteries following balloon angioplasty were among the first to suggest that TGFβ plays a prominent role in the development of IH following revascularisation surgery. These studies showed that the mRNA expression of TGFβ1 was significantly elevated in restenotic lesions compared with both primary atherosclerotic lesions and control non-atherosclerotic tissues [107]. IHC co-staining for TGFβ1 and αSMA within sections taken adjacent to those studied by *in situ* hybridisation demonstrated that TGFβ1 was localised to the intimal and medial SMC layers in restenotic lesions [107]. Two further groups demonstrated that the majority of human restenotic lesions showed positive staining for TGFβ1 throughout the vascular media and intima, identifying SMCs as a key source of TGFβ1 in restenotic vessels [108,109]. Pre-clinical studies support this observation, showing that TGFβ1 is upregulated in SMCs at sites of acute vascular injury in rodents. TGFβ1 mRNA levels were significantly increased in rat carotid arteries 6 hours post-injury and remained significantly elevated for at least 2 weeks [110,111]. Increased TGFβ1 expression (both at the mRNA and protein level) 2 weeks after wounding was associated with a parallel increase in fibronectin, collagen I and collagen III mRNA expression, three pro-fibrotic genes known to be regulated by TGFβ [111]. In a porcine coronary angioplasty model, levels of active TGFβ1 were significantly elevated between 2 hours and 7 days following angioplasty, with immunohistochemical studies showing strong localisation to both SMCs and ECs [112]. TGFβ1 mRNA and protein levels have also been shown to be chronically upregulated 6 months post-grafting in a rabbit CABG model, accompanied by increases in connective tissue growth factor (CTGF), a well-defined TGFβ1 responsive gene [113].

Subsequently, numerous *in vivo* interventional studies have convincingly shown that TGFβ promotes IH in vein grafts and models of PCI (Table 2). Overexpression of TGFβ1 in porcine arteries using an expression plasmid resulted in increased procollagen, collagen and proteoglycan synthesis by neointimal SMCs and was accompanied by marked intimal and medial hyperplasia [114]. Furthermore, adenoviral overexpression of active TGFβ1 in uninjured rat arteries resulted in a hyperplasic neointima [96] or a larger collagen-rich neointima in grafted rat arteries compared with control [115]. Similarly, infusion of purified recombinant TGFβ1 into rats 2 weeks after carotid artery injury increased [3H]-thymidine-labelled SMC nuclei within the neointima, indicating that TGFβ1 stimulates SMC proliferation as well as collagen synthesis in this model of vascular injury [111]. Substantiating these TGFβ1 overexpression studies, inhibition of TGFβ by antisense treatment or by intravenous infusion of a soluble TβRII reduced IH and adventitial fibrosis in balloon-injured rat carotid arteries [116,117]. Interestingly, adenovirus-mediated overexpression of TGFβ3 (but not TGFβ1) in pig coronary arteries inhibited constrictive remodelling and reduced lumen loss after coronary angioplasty [118]. Similarly, direct infusion of TGFβ3 to goat carotid arteries after anastomosis reduced vessel wall thickness by 30%, in part by reducing collagen type VII content 3 months post-surgery [119]. In contrast to the increased intimal hyperplasia observed in interventional studies using TGFβ1, these groups also showed reduced SMC proliferation in TGFβ3-treated animals, which suggests that there may be differences in how SMCs respond to specific TGFβ isoforms *in vivo*. The intracellular signalling mechanisms that drive IH in response to TGFβ have yet to be fully characterised, however the majority of studies to date have identified an important role for the canonical Smad2/3 pathway. Kundi *et al* showed that carotid artery injury in rats leads to significant induction of Smad3 in medial SMCs, while overexpression of Smad3 via gene transfer resulted in increased collagen accumulation [120] and SMC proliferation via a p27-dependent mechanism [97]. Furthermore, adenoviral overexpression of Smad7 in rat balloon-injured arteries reduced intimal thickening, lumen area loss and collagen synthesis 14 days post-injury [121], with *in vitro* studies indicating that these effects were due to direct effects on resident SMCs [122,123]. Interestingly, oral dosing of an ALK5/ALK4 small molecule inhibitor following balloon injury decreased intimal collagen production but had no effect on intimal SMC proliferation [124], suggesting that other TGFβ receptor signalling pathways may be responsible for TGFβ-induced SMC proliferation.

**Table 2:** *In vivo* studies employing different approaches to target TGFβ activity after vascular injury

Therapy	Animal model	Outcome compared to control	Reference
Soluble TβRII	Rat carotid artery balloon injury	Reduced intimal thickening, constrictive remodelling, lumen area loss and collagen type I/III mRNA expression	Smith et al, 1999 [116]
ALK4/5/7 inhibitor (SB431542)	Rat carotid artery balloon injury	Reduced intimal thickening, neointimal SMC proliferation, reduced recruitment of MSCs	Zhao et al, 2016 [182]
ALK4/5 kinase inhibitor (SM16)	Rat carotid artery balloon injury	Reduced intimal thickening, inhibition of adventitial myofibroblast formation, collagen deposition	Fu et al, 2008 [124]
Anti-TGFβ1 ribozyme oligonucleotides	Rat carotid artery balloon injury	Reduced intimal thickening, TGFβ1 mRNA expression, collagen type I/III expression and synthesis	Yamamoto et al, 2000 [117]
Anti-TGFβ1 phosphorothioate oligonucleotides	Rabbit carotid artery balloon injury	Reduced intimal thickening, proteoglycan synthesis and TGFβ1 mRNA expression	Merrilees et al, 2000 [183]
Tranilast	Rat carotid artery balloon injury	Reduced SMC migration, TGFβ1 mRNA expression, TβRI/TβRII mRNA expression and αVβ3 mRNA expression	Ward et al, 1998 [184]
TGFβ1 antisense mRNA (adenoviral overexpression)	Rat femoral artery vein grafting	Reduced intimal thickening, reduced collagen and TIMP mRNA expression	Wolff et al, 2006 [115]
Recombinant TGFβ3	Pig coronary artery balloon injury	Reduced constrictive remodelling, lumen area loss and increased collagen synthesis	Kingston et al, 2003 [118]
Smad7 (adenoviral overexpression)	Rat carotid artery balloon injury	Reduced intimal thickening, lumen area loss, collagen synthesis and adventitial fibroblast migration	Maallawaarachchi et al, 2005
p38 MAPK inhibitors	Rat carotid artery balloon injury	Reduced intimal thickening and SMC proliferation	Ohashi et al, 2000 [185]
Pyrrole-imidazole polyamide targeting the TGFβ1 promoter	Rat carotid artery balloon injury	Reduced intimal thickening, TGFβ1, collagen and fibronectin mRNA expression and accelerated re-endothelialisation	Yao et al, 2009 [186]

TGFβ = transforming growth factor beta, BMP = bone morphogenetic protein, GDF = growth/differentiation factor, AMH = anti-Mϋllerian hormone, RGM = repulsive guidance molecule

## 6 TGFβ signalling and SMC function

As stated in the introduction, studies have conclusively shown that TGFβ is a potent regulator of SMC phenotype and function. The atheroprotective effects of TGFβ are in part attributed to its capacity for stimulating SMC differentiation by inducing the expression of a large set of mature SMC genes (including αSMA, SM22α and SMMHC [125]) via Smad2 and/or Smad3, which interact with the SMC-specific promoters at putative SBEs [126,127]. TGFβ also induces serum response factor (SRF) protein expression and enhances its binding activity to CArG elements within the promoters of SMC marker genes [128]. Interestingly, Qiu et al have shown that Smad3 is the primary mediator for TGFβ1-induced SM22α expression, while Smad6 and Smad7 repress its activation [129]. Furthermore, the authors illustrated that Smad3 can bind to a SBE in the first exon of SM22α and directly associate with the SRF complex in response to TGFβ1 treatment [129]. TGFβ is also a potent inducer of the synthetic SMC phenotype, stimulating the production and secretion of collagen and proteoglycans by SMCs via direct and indirect interactions with the promoters of these genes [[130], [131], [132]]. However, the effects of TGFβ on SMC behaviour are more variable, with studies showing that TGFβ can both inhibit and stimulate SMC proliferation and migration. This may be due to the heterogeneous nature of SMCs, as evidenced by the varying gene expression patterns of human SMCs derived from primary atherosclerotic plaques, in-stent stenoses or healthy arteries [133]. At the molecular level, these differences have been attributed to varying levels of receptor expression, membrane localisation of receptors, availability of intracellular signalling mediators and presence of transcriptional co-regulators within the nucleus (reviewed in [54,134]). In the next section, we will highlight key findings on the regulation of SMC proliferation and migration by TGFβ in the context of intimal hyperplasia and CAD.

## 7 TGFβ-regulated SMC proliferation

SMC responses to TGFβ *in vitro* are influenced by factors such as type of SMC (aortic, venous etc.), cellular density and concentration of TGFβ [134]. For example, Majack et al found that TGFβ1 inhibited proliferation of rat aortic SMCs at sub-confluent densities but potentiated SMC growth at high seeding densities [135]. Furthermore, treatment of cultured porcine coronary artery SMCs with low concentrations of TGFβ1 (0.025ng/mL) stimulated SMC proliferation, but attenuated SMC growth at concentrations of greater than 0.1 ng/mL [136]. The presence of other growth factors also appears to influence the effects of TGFβ on SMC proliferation. For instance, treatment of rat aortic SMCs with TGFβ1 had no significant effect on cell number in quiescent SMC cultures maintained in 1 % FBS, but markedly inhibited SMC proliferation in response to 5 % FBS or PDGF-BB in a dose-dependent manner [135,137]. Other studies, however, have shown that TGFβ potentiates the mitogenicity of FBS, PDGF-BB and bFGF, but only in confluent SMC cultures [138,139].

TGFβ-induced inhibition of SMC proliferation *in vitro* has been associated with G0/G1 cell cycle arrest through downregulation of the cell cycle regulator, cyclin-dependent kinase 1 (CDK1) [140]. Treatment of mouse aortic SMCs with TGFβ1 for 24 hours substantially reduced the percentage of cells in S phase and G2/M phase and increased the number of cells in G0/G1 [141]. Pharmacological inhibition of the p38 MAPK pathway (using 10μM SB203580) resulted in complete attenuation of TGFβ-dependent growth inhibition in the absence of any inhibitory effect on Smad2/3 signalling, as analysed by phosphorylation, nuclear translocation and reporter gene expression (141), indicating that p38 MAPK may mediate growth inhibition induced by TGFβ in SMCs. More recently, TGFβ has been shown to inhibit PDGF-induced SMC proliferation through downregulation of Cyclin D1 [142], a key regulator of cell cycle transition from G1 to S phase [143]. Here the authors demonstrated that treatment of human aortic SMCs with TGFβ1 significantly inhibited PDGF-BB-induced Cyclin D1 mRNA and protein expression after 24 hours. Interestingly, inhibition of ALK5 using 10 μM SB431542 or siRNA-mediated knockdown of Smad4 completely abolished the inhibitory effect of TGFβ on PDGF-induced Cyclin D1 expression and restored SMC proliferation in response to PDGF, suggesting that this occurs through a Smad-dependent mechanism [142].

In contrast, certain studies have shown that TGFβ1 can indirectly promote SMC proliferation in confluent cultures by inducing PDGF-A gene expression and autocrine production of PDGF-AA [138,144]. Both these studies found that TGFβ-induced rat aortic SMC proliferation was mimicked by treatment with exogenous PDGF-AA (> 5 ng/ml) and partially inhibited by neutralising antibodies to PDGF-AA [138,144]. However, a later study showed that while TGFβ induced an 8-fold increase in PDGF concentration after 24 hours, application of this conditioned medium (containing ~ 1 ng/mL PDGF-AA) to aortic SMCs did not increase mitogenic activity, indicating that induction of PDGF-AA production by TGFβ cannot fully account for the effects of TGFβ on the proliferation of rat aortic SMCs under all *in vitro* culture conditions [145]. Indeed, TGFβ has also been shown to directly stimulate SMC proliferation through a Smad-dependent mechanism. For instance, Mao et al demonstrated that aortic SMCs from smooth muscle-specific Smad4 knockout mice display a 62 % reduction in proliferation *in vitro* (as determined by BrdU labelling), compared with SMCs from wild-type mice [146]. Furthermore, shRNA-mediated knockdown of Smad2 and Smad3 within wild-type SMCs significantly reduced SMC proliferation in response to 20 % FBS and the expression of SMC-specific marker genes [146].

Despite the contrasting *in vitro* data for the effects of TGFβ on SMC proliferation, the majority of *in vivo* evidence indicates that TGFβ is a potent stimulator of arterial SMC proliferation [96,97,111,147]. For instance, infusion of recombinant TGFβ1 into rats after carotid artery balloon injury resulted in a significant increase in the number of [3H]-thymidine labelled SMC nuclei within the neointima, compared with untreated rat coronary arteries [111]. Similarly, Schulick et al noted that localised adenoviral over-expression of TGFβ1 in the endothelium of uninjured rat carotid arteries resulted in substantial intimal thickening after 4 weeks with marked cellular proliferation (measured by BrdU incorporation) when compared with control arteries [96]. TGFβ-induced SMC proliferation *in vivo* has been shown to be mediated via a Smad3-dependent mechanism, involving the phosphorylation and nuclear export of the cyclin-dependent kinase inhibitor p27 [97]. Adenoviral overexpression of Smad3 within balloon-injured rat carotid arteries significantly enhanced intimal thickening after 14 days and was associated with increased PCNA expression within intimal SMCs [97] and increased pERK MAPK expression within whole arteries and isolated SMCs [147]. Conflicting studies performed using a more damaging, inflammatory model of femoral artery wire injury showed enhanced neointimal hyperplasia and increased SMC proliferation in Smad3 knockout mice, indicating that the role of TGFβ in the arterial response to injury can vary as a function of the inflammatory microenvironment [148]. Thus, TGFβ/Smad3 can directly enhance SMC proliferation *in vivo* through transactivation of the ERK MAPK signalling pathway; other indirect mechanisms may account for the enhanced or repressed proliferative responses observed, including modulation of the inflammatory microenvironment or release of sequestered mitotic growth factors following ECM degradation.

## 8 TGFβ-regulated SMC migration

Similar to SMC proliferation, TGFβ has been shown to variably stimulate and inhibit SMC migration. Early *in vitro* studies performed in venous and arterial-derived SMCs showed that PDGF-BB, b-FGF or serum-induced migration is inhibited by TGFβ1 in a concentration-dependent manner and this effect is independent of cellular density [149,150]. TGFβ1 can suppress PDGF-BB-induced up-regulation of MMP-2 within rat arterial SMCs, suggesting that the indirect effects of TGFβ1 on SMC migration may partly be due to the inhibition of downstream pro-migratory genes [151]. Conversely, studies also show that TGFβ can directly stimulate SMC migration. For instance, aortic SMCs from smooth muscle-specific Smad4 knockout mice displayed significantly reduced migration in response to serum or PDGF-BB *in vitro*, compared with SMCs from wild-type mice [146]. Furthermore, inhibition of ALK5 using the kinase inhibitor SB431542 or shRNA-mediated knockdown of Smad2 or Smad3 significantly attenuated SMC migration in response to serum stimulation [146]. *In vitro* studies performed on aortic SMCs have shown that TGFβ can also regulate SMC migration via indirect mechanisms involving the up-regulation of avβ3 mRNA expression, an integrin which is highly expressed following vascular injury and is important in driving SMC migration [[152], [153], [154], [155]]. Pre-treatment of human aortic SMCs with TGFβ1 was associated with enhanced migration in response to vitronectin, a serum glycoprotein which promotes cell spreading and attachment through integrin receptor binding [154]. Furthermore, treatment of injured rat carotid artery SMCs with a TGFβ1 neutralising antibody completely abrogated TGFβ1-induced integrin β3 mRNA up-regulation [156]. Interestingly, treatment of rats with genistein (a tyrosine kinase inhibitor) following carotid artery injury markedly inhibited injury-induced up-regulation of TGFβ1, TGFβ3, integrin av and β3 mRNA expression, compared with vehicle-treated arteries, suggesting that induction of TGFβ following vascular injury is broadly reliant on tyrosine kinases [156].

## 9 Therapeutic targeting of TGFβ in CAD: challenges and opportunities

As documented above, TGFβ plays a fundamental role in the regulation of vascular function by affecting SMC proliferation, migration, differentiation and ECM production in CAD. Mutations in genes encoding TGFβ ligands and receptors are also associated with several developmental disorders and vascular diseases, including Marfan syndrome type 2, Loeys-Dietz syndrome, and other vasculopathies with clinical presentations that include thoracic aortic aneurysms and dissections [[157], [158], [159]]. Hence, components of the TGFβ signalling pathway are important therapeutic targets for a wide range of vascular pathologies.

Numerous pre-clinical studies have employed different approaches to inhibit TGFβ signalling after vascular injury, which have been shown to reduce intimal thickening compared with controls. However these approaches have yet to translate to significant clinical gain in the cardiovascular disease arena, with no TGFβ therapeutics currently on the market. Promisingly, small-scale clinical trials demonstrated that oral administration of 600 mg/day tranilast (N-(3,4-dimethoxycinnamoyl) anthranilic acid), a non-specific inhibitor of TGFβ biosynthesis, was associated with a significantly reduced risk of restenosis following PCI, compared with placebo (17.6% vs. 39.4% at 3 months) [160,161]. Originally developed as a treatment for allergic disorders such as chronic rhinitis and bronchial asthma, tranilast has also successfully been used (both orally and topically) as an anti-fibrotic agent in the treatment of hypertrophic scars or keloids [[162], [163], [164]]. However, the large-scale randomised double-bind clinical trial PRESTO (Prevention of REStenosis with Tranilast and its Outcomes) examining the effects of tranilast treatment in 11,484 patients after PCI failed to show improved clinical outcome (death, MI or repeat revascularisation) compared with placebo [165]. Worryingly, this trial highlighted some potential adverse effects of tranilast, including hyperbilirubinemia, increased serum creatinine and alanine transaminases, indicative of liver abnormalities. Fortunately these adverse effects were reversed upon cessation of treatment, however the lack of primary and secondary endpoint efficacy in this large-scale trial highlights the complexity of targeting TGFβ using systemic approaches in multimorbid, highly diverse groups of patients.

Nevertheless, TGFβ therapeutics are advancing in clinical trials for other indications, particularly fibrosis and oncology, and results appear to be positive [166,167]. Indeed, Pirfenidone (5-methyl-1-phenyl-2-[1*H*]-pyridone), which inhibits TGFβ production and activity, was approved by the FDA in October 2014 for treatment of idiopathic pulmonary fibrosis (IPF). IPF is a devastating progressive lung disease, with a median survival from time of diagnosis of 3 years; Pirfenidone was approved on the basis of phase III clinical trials showing a reduction in forced vital capacity decline (a measure of lung function) and improved progression-free survival compared to placebo (ASCEND study [168]). In the oncology field, Galunisertib (LY2157299 monohydrate) a small molecule inhibitor of the ALK5 kinase, has been evaluated in >10 clinical trials (alone or in combination with e.g alkylating agents) for different types of cancer [169]. The most advanced trial currently in progress is a phase II/III randomised, placebo-controlled trial that has enrolled ~140 patients with myelodysplastic syndrome (MDS; NCT02008318); interim data from this trial shows good tolerance of the drug and haematological improvement in 26% of patients enrolled. Of note, recent trials investigating the use of Galunisertib have utilised an adapted, intermittent dosing regimen (14 days on, 14 days off) due to preclinical studies showing proliferative, inflammatory changes in the heart valves and aortae of rats when continuously dosed with Galunisertib [170]. Although no medically significant cardiotoxicities were observed in a first-in-human dose study administering Galunisertib to glioma patients [171], the potential for serious adverse events with high-dose, systemic TGFβ agonists or antagonists should not be underestimated. Ultimately, localised and pathway-specific targeting of TGFβ signalling will be required in order to achieve optimal therapeutic efficacy whilst avoiding undesired off-target effects.

While there are acknowledged challenges associated with using global approaches for targeting TGFβ in multimorbid CAD patients, new avenues with the potential for more focused targeting of TGFβ in SMCs have recently opened up. In the last decade, next-generation sequencing studies have identified non-coding RNA (ncRNA) sequences residing in intergenic regions of the genome. These non-coding transcripts are now known to have multiple functions, regulating the transcription and translation of proximal and distant protein-coding genes in a context-specific manner (reviewed in [172]). Recent studies have begun to elucidate the interactions between them and TGFβ pathway components, identifying novel potential therapeutic targets for CAD. Early studies showed that TGFβ could alter the expression of numerous microRNAs (miRs) in various human tissues and cells, the effects of which appear to be cell-type specific [173]. Microarray analysis in human carotid artery SMCs revealed a number of differentially expressed miRs following TGFβ1 treatment, including miR-143/145, which was significantly up-regulated by TGFβ1 in a concentration- and time-dependent manner [174]. Treatment of SMC with a specific inhibitor of p38MAPK completely blocked TGFβ1-induced miR-143/145 expression and attenuated the expression of SMC contractile genes (including CNN1, TAGLN and ACTA2) in response to TGFβ1 stimulation [174], identifying an additional mechanism through which TGFβ1 can promote SMC differentiation. Interestingly the miR-143/145 cluster, which is highly enriched in SMCs, has been shown to be significantly decreased following acute arterial injury [175] and in mouse atherosclerotic lesions [175]. Genetic knockout of miR-143/145 led to a reduction in the number of contractile arterial SMCs and a corresponding increase in synthetic SMCs, as determined by electron microscopy [176]. Neointimal lesions were also frequently observed in the femoral arteries of aged miR-143/145^-/-^ mice, with no lesions observed in wild-type animals [176]. TGFβ has also been shown to regulate the expression of miR-21 through promoting the processing of pri-miR-21 into pre-miR-21 by the Drosha complex [177]. Importantly, miR-21 is over-expressed in murine and porcine models of vein grafting and is highly expressed within αSMA+ SMCs of failed human vein grafts [178]. Genetic ablation or antisense oligonucleotide-mediated knockdown of miR-21 significantly attenuated injury-induced neointima formation by inhibiting SMC proliferation and migration and inducing SMC apoptosis, highlighting the potential therapeutic benefit of miR-21 inhibition [178,179]. Together, these studies indicate that TGFβ-regulated miRNAs play a critical role in controlling SMC phenotype transitions and the response of the vascular wall to injury, underlining their potential as therapeutic targets. Targeting SMC-enriched, disease-dysregulated miRs downstream of TGFβ may be a more rational approach for achieving therapeutic efficacy whilst avoiding undesired side-effects.

## 10 Concluding remarks

TGFβ was initially identified in the early 1980’s, when Anita Roberts and Michael Sporn purified a ‘transformation factor’ that could render healthy cells malignant [180]. The first observation that this Janus-like cytokine could have multifunctional effects was made shortly thereafter, in studies showing that TGFβ could synergise with PDGF to stimulate fibroblast colony formation (CF) whilst inhibiting epidermal growth factor-induced CF [181]. From these early beginnings, the field of TGFβ research – and indeed the TGFβ superfamily - has expanded exponentially, with papers on TGFβ now numbering in the tens of thousands. Nevertheless, important questions have yet to be fully answered, and our understanding of the many TGFβ paradoxes remains incomplete. The advent of next-generation sequencing (NGS) has provided some clarification, identifying hitherto unknown genetic and phenotypic overlaps between patients who develop cardiovascular disease and those with inherited vascular conditions caused by mutations in TGFβ genes. Alongside, investigations following on from the Human Genome Project have started unravelling the complexity of the transcriptome, identifying non-coding RNA sequences that both regulate and are regulated by TGFβ signalling. These and other studies have greatly enhanced our mechanistic understanding of TGFβ, and the many levels at which this pleiotropic cytokine is controlled. From early experiments showing that TGFβ enhances the secretion of ECM proteins, we are now beginning to grasp how the cellular microenvironment in turn influences the actions of TGFβ; this is of particular relevance to coronary artery disease and intimal hyperplasia, during which extensive vascular remodelling occurs. Elucidation of these and other questions regarding the actions and interactions of TGFβ will, we hope, lead to the development of localised and pathway-specific therapies that effectively and selectively target the pathological actions of TGFβ.

## Sources of funding

Dr. Low is supported by a British Heart Foundation PhD Studentship (FS/12/66/30003), Prof. Baker is supported by the British Heart Foundation Chair of Translational Cardiovascular Sciences (CH/11/2/28733) and Dr. Bradshaw is supported by a Personal Research Fellowship from the Royal Society of Edinburgh (RSE/33457).
